# The Neonatal-Perinatal Medicine fellowship application webinar series: a novel approach to helping applicants succeed

**DOI:** 10.1186/s12909-023-04644-z

**Published:** 2023-09-18

**Authors:** Michelle J. Bartlett, Ellen Ribar, Yingying Zheng, Sasha Amiri, Nicolle Fernández Dyess, Ashley M. Lucke, Heather French

**Affiliations:** 1 Department of Pediatrics, Section of Neonatology, Children’s Hospital of Philadelphia, Philadelphia, USA; 2 Department of Pediatrics, Section of Neonatology, University of Colorado, Aurora, USA; 3 Department of Pediatrics, Section of Neonatology, East Carolina University, Greenville, USA; 4 Department of Pediatrics, Section of Neonatology, Rush University Medical Center, Chicago, USA; 5 Department of Pediatrics, Section of Neonatology, Dell Children’s Medical Center, Pediatrix Medical Group, University of Texas at Austin, Austin, USA

**Keywords:** Neonatal-Perinatal Medicine fellowship application, Webinar series, Fellowship preparation

## Abstract

**Background:**

In 2022, 13,586 candidates applied to subspecialty fellowships. Formal resources to inform candidates on subspecialty-specific fellowship application are limited. Candidates rely on residency application experience, informal advice, and online research for navigating the application process. Thus, a need exists for formal subspecialty-specific fellowship application guidance. The American Academy of Pediatrics Organization of Neonatal-Perinatal Medicine Training Program Directors (ONTPD) and Trainees and Early Career Neonatologists (TECaN) created a webinar-based curriculum to help educate trainees about the application process and recruit diverse fellowship applicants.

**Methods:**

In 2022, ONTPD and TECaN co-hosted and implemented a four-part national webinar series focused on different aspects of the Neonatal-Perinatal Medicine (NPM) fellowship application and interview processes. Webinars were advertised through list-servs and social media, conducted in two time zones, and recorded for asynchronous viewing. Registration, demographic data, and questions for webinar panelists were collected via electronic survey. Program evaluation data was collected after each webinar and following the fellowship match.

**Results:**

In the 2022 appointment year, 310 candidates participated in the NPM fellowship match and 250 individuals registered for the webinar series. A quarter (26%) of registrants identified as underrepresented in medicine. Most registrants reported minimal or no knowledge of the fellowship application (64%, 158/248) and interview (81%, 201/248) processes. The majority of registrants (70%, 173/248) were planning on applying to fellowship in 2022, and 91% of post-webinar respondents (43/47) felt the webinars were moderately or extremely helpful, a finding that was sustained beyond the match (37/42). Almost all respondents rated the quality of the webinars as good or higher and were likely or very likely to recommend them to peers (39/42). There was considerable variability amongst respondents in the number of fellowship programs applied to, interviewed at, and ranked, and factors influencing rank list.

**Conclusion:**

We describe a virtual curriculum to prepare trainees for the NPM fellowship application and interview process. This webinar series provides needed education to fellowship candidates, bridges the gap between candidate knowledge and program director expectations, is generalizable to other specialties, and can be replicated with minimal resources.

## Background

In 2022, 13,586 candidates applied for subspecialty fellowships, making it the largest application cycle in the history of the National Resident Matching Program (NRMP) Specialties Matching Service (SMS) [1]. The fellowship application process varies between subspecialties, and limited subspecialty-specific data and guidance on application preparation exists. Unfamiliarity with how to make written applications reflect commitment and passion for a field and lack of knowledge about and preparation for interviews can impact an applicant’s future career. Additional uncertainties for applicants include the number of programs to apply to, subspecialty-specific trainee expectations, and identification of fellowship programs whose expertise aligns with the applicant’s scholarly interests. In addition, with the transition to the virtual interview format, applicants may be less familiar with the processes and expectations of interviewing virtually [2].

There are numerous resources and support for medical students making the transition to residency; however, there are fewer national discussions and resources supporting residents transitioning to fellowship [3–5]. There is evidence showing medical students appreciate residency preparation courses (RPCs) during their 4th year of medical school, indicating that trainees appreciate transition-focused curriculum [6]. There are no current needs assessment studies in the literature to support that residents transitioning to fellows would appreciate similar structured guidance, which identifies a gap in knowledge in trainees’ attitudes towards the fellowship application and transition process.

Further, it is known that there is systemic bias and inequity in how residency programs review and select trainees because there can be an emphasis on inequitable measurement tools such as Alpha Omega Alpha (AΩA), clerkship grades, and the United States Medical Licensing Examination (USMLE) scores [3, 7, 8]. As fellowship programs consider where a candidate has completed residency training in selecting their own trainees, systemic biases and inequities in each transition of training likely have downstream and compounding effects affecting fellowship training as well [3]. URiM trends in Neonatal-Perinatal Medicine (NPM) have shown a decline in representation in recent years despite unchanged representation in pediatric residencies [9]. In order to reduce disparities in the fellowship selection process, applicants need to have transparent equal access resources in addition to demonstrated focus and diverse representation from NPM fellowship programs.

We aimed to develop a novel webinar-based NPM-specific fellowship application curriculum with guidance from NPM program directors and national organizations. Objectives of the curriculum included creating a free resource with diverse representation from numerous NPM fellowships, reaching a large and diverse applicant pool, and increasing applicants' knowledge of the NPM fellowship application and virtual interview process. We hypothesized that the webinar-based curriculum would reach our target population and increase their knowledge of the NPM fellowship application and interview process.

## Methods

### Webinar development

From May to July 2022, the American Academy of Pediatrics (AAP) Organization of Neonatal-Perinatal Medicine Program Directors (ONTPD) and Trainees and Early Career Neonatologists (TECaN) co-hosted six virtual webinars via Zoom (Zoom Video Communication, Inc.) spanning four content areas (Table 1). Topics focused on the NPM application and interview process from the program leadership perspective in addition to specific webinars for experiential advice from fellows and physician-scientists. These topics were selected to maximize transparency and dissemination of relevant information aimed at reaching a diverse group of trainees interested in NPM fellowship. Webinars were scheduled for sixty minutes in the evening hours across different time zones to facilitate broad participation and were recorded for asynchronous viewing on TECaN’s YouTube channel. Webinar advertisement occurred through professional networks, subspecialty list-servs, and social media platforms. Each webinar had five to seven panelists of NPM fellowship program directors/associate program directors (PDs), fellows, or physician-scientists, as appropriate for the content. The webinar planning committee utilized professional networks to select panelists that maximized diversity of gender, ethnicity, and fellowship program location and size. Panelists were provided with committee-selected and registrant-submitted questions in advance. Two to three panelists answered each question. Synchronous attendees could also ask questions in real-time via the webinar chat feature.

**Table 1:** Composition and attendance of webinar series during the 2022–2023 academic year

	**# of Panelists**	**Live Attendance**	**Online views**
**Mastering the NPM Fellowship Application**			
Eastern Time Zone PD Panel	5	99	125
Western Time Zone PD Panel	5	31	125
**Mastering the NPM Fellowship Interview**			
Eastern Time Zone PD Panel	5	30	149
Western Time Zone PD Panel	5	54	100
**NPM Fellows Panel**	7	45	107
**NPM Physician Scientist Panel**	7	22	47

### Study design

Survey data was collected via Research Electronic Data Capture (REDCap) [10]. A total of 6 surveys were sent to registrants and included: webinar registration survey, individual post-webinar evaluation surveys, and a final post-match survey. The registration survey was not linked to demographic and evaluation data collection to preserve anonymity. Participants registered for the webinars in order to obtain access to the webinar Zoom link. Survey questions included select-all, multiple-choice, Likert-scale questions, and free response. Each survey was designed to take less than 10 min. Demographic data, experience and comfort with the fellowship application process, as well as questions for panelists were collected via the webinar registration survey. At the end of each webinar, participants were prompted to complete a post-webinar evaluation survey on the quality and utility of the specific webinar via QR code and link in the webinar chat. In addition, a final post-match survey encompassing overall evaluation of the webinar series and collection of post-match outcomes was distributed via email to all webinar registrants. The final survey assessed the overall helpfulness of the webinar series to improve knowledge of the application and interview process, impact on fellowship matching, and asked for feedback to improve future webinars. All methods were performed in accordance with the Declaration of Helsinki, the study was approved by the ethics committee of University of Colorado’s Institutional Review Board, and informed consent was obtained from all subjects.

### Target audience

Participants included current pediatric residents and post-residency physicians who were interested in learning more about the NPM fellowship application and interview processes.

### Statistical analysis

Survey data was collected and stored in REDCap hosted at The Children’s Hospital of Philadelphia. Participant survey data was analyzed utilizing Excel and REDCap software. Descriptive statistics were used to analyze the quantitative data.

## Results

A total of 250 registrants and 34 panelists participated in the webinar series. A total of six webinars, spanning four content areas were held (Table 1). Fellowship PDs and associate PDs were panelists for webinars on building a competitive Electronic Residency Application Service (ERAS) NPM application (“Mastering the NPM Fellowship Application”) and virtual fellowship interview best practices (“Mastering the NPM Fellowship Interview”). Current NPM physician-scientists provided career options and advice to aspiring NPM physician-scientists in the “NPM Physician Scientist Panel”. Current NPM fellows provided experiential advice to fellowship applicants in the “NPM Fellows Panel”. The majority of registrants learned about the series from local faculty members or their residency program.

**Fig. 1:**
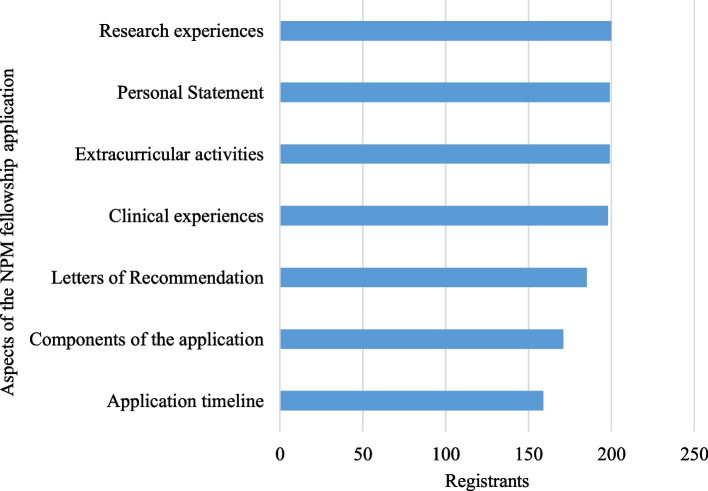
Specific aspects of the NPM fellowship application that registrants were interested in learning about collected from our registration survey

Most registrants were first- and second-year residents from diverse geographic areas, and 26% self-identified as URiM (Table 2). Most participants (70%) were planning on applying to fellowship during the 2022 application cycle, yet the majority reported minimal to no knowledge of fellowship application (63%) and interview (80%) processes (Table 2). In addition, a majority of our participants wanted to learn about a variety of aspects of the NPM fellowship application process (Fig. 1). The largest live audience was 99, combining all of the webinars, we had over 650 online views (Table 1). A majority of those completing the post-match survey stated they were likely or very likely to recommend these webinars to a peer or colleague (93%) and felt the webinars were moderately or extremely helpful (88%) (Table 3). In addition, all of the post-match survey respondents rated the quality of the webinars as good or higher (Table 3).

**Table 2:** Demographic information of registrants

Registrant Information	N (%)
Level of Training (*N* = 246)	
*PGY-1*	50 (20%)
*PGY-2*	132 (53%)
*PGY-3*	48 (19%)
*PGY-4*	0 (0%)
*PGY-5* +	4 (2%)
*Completed Residency*	12 (5%)
Gender Identity (*N* = 240)	
*Female*	197 (79%)
*Male*	41 (16%)
*Prefer to not answer*	2 (1%)
Identifies as Underrepresented in Medicine (URiM) (*N* = 239)	
*Yes*	65 (26%)
*No*	174 (73%)
Location of residency program (*N* = 249)	
*South Atlantic (DC, DE, FL, GA, MD, NC, SC, VA, WV)*	64 (26%)
*Mid Atlantic (NJ, NY, PA)*	62 (25%)
*East North Central (IL, IN, MI, OH, WI)*	40 (16%)
*Pacific (AK, CA, HI, OR, WA)*	33 (13%)
*New England (CT, MA, ME, NH, RI, VT)*	15 (6%)
*East South Central (AL, KY, MS, TN)*	14 (5%)
*West South Central (AR, LA, OK, TX)*	14 (5%)
*West North Central (IA, KS, MN, MO, ND, NE, SD)*	10 (4%)
*Mountain (AZ, CO, ID, MT, NM, NV, UT, WY)*	5 (2%)
*Territory (PR)*	2 (1%)
Knowledge level regarding the NPM fellowship application (*N* = 247)	
*Not at all*	71 (28%)
*Slightly knowledgeable*	87 (35%)
*Somewhat knowledgeable*	66 (26%)
*Moderately knowledgeable*	22 (9%)
*Extremely Knowledgeable*	1 (0%)
Knowledge level regarding NPM Fellowship Interviews (*N* = 247)	
*Not at all*	113 (45%)
*Slightly knowledgeable*	88 (35%)
*Somewhat knowledgeable*	41 (16%)
*Moderately knowledgeable*	4 (2%)
*Extremely Knowledgeable*	1 (0%)
Applying for NPM fellowship in academic year 2022–2023 (*N* = 248)	
*Yes*	173 (70%)
*No*	67 (27%)
*Unsure*	8 (3%)

**Table 3:** Post-match survey data-program evaluation outcomes

Program Evaluation Outcomes	N(%)
Helpfulness of the webinar series (*N* = 42)	
*Not at all helpful*	0 (0%)
*Slightly helpful*	1 (2%)
*Somewhat helpful*	4 (10%)
*Moderately helpful*	13 (31%)
*Extremely helpful*	24 (57%)
Quality of the webinar series (*N* = 42)	
*Poor*	0 (0%)
*Fair*	0 (0%)
*Good*	6 (14%)
*Very good*	19 (45%)
*Excellent*	17 (40%)
How likely registrants were to recommend the webinar series to peer or a colleague (*N* = 42)	
*Very unlikely*	0 (0%)
*Unlikely*	0 (0%)
*Neutral*	3 (7%)
*Likely*	10 (24%)
*Very likely*	29 (69%)

**Fig. 2:**
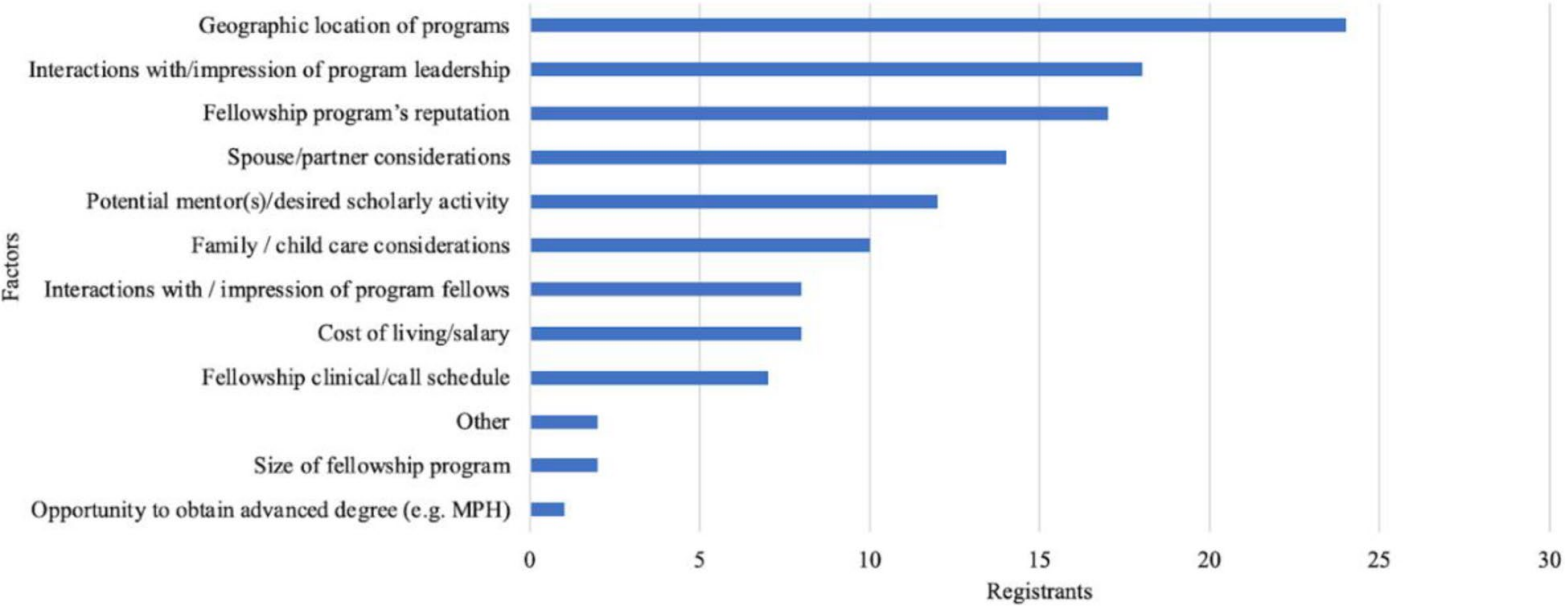
Specific factors that influenced candidates ranking programs collected from our post-match survey

**Fig. 3:**
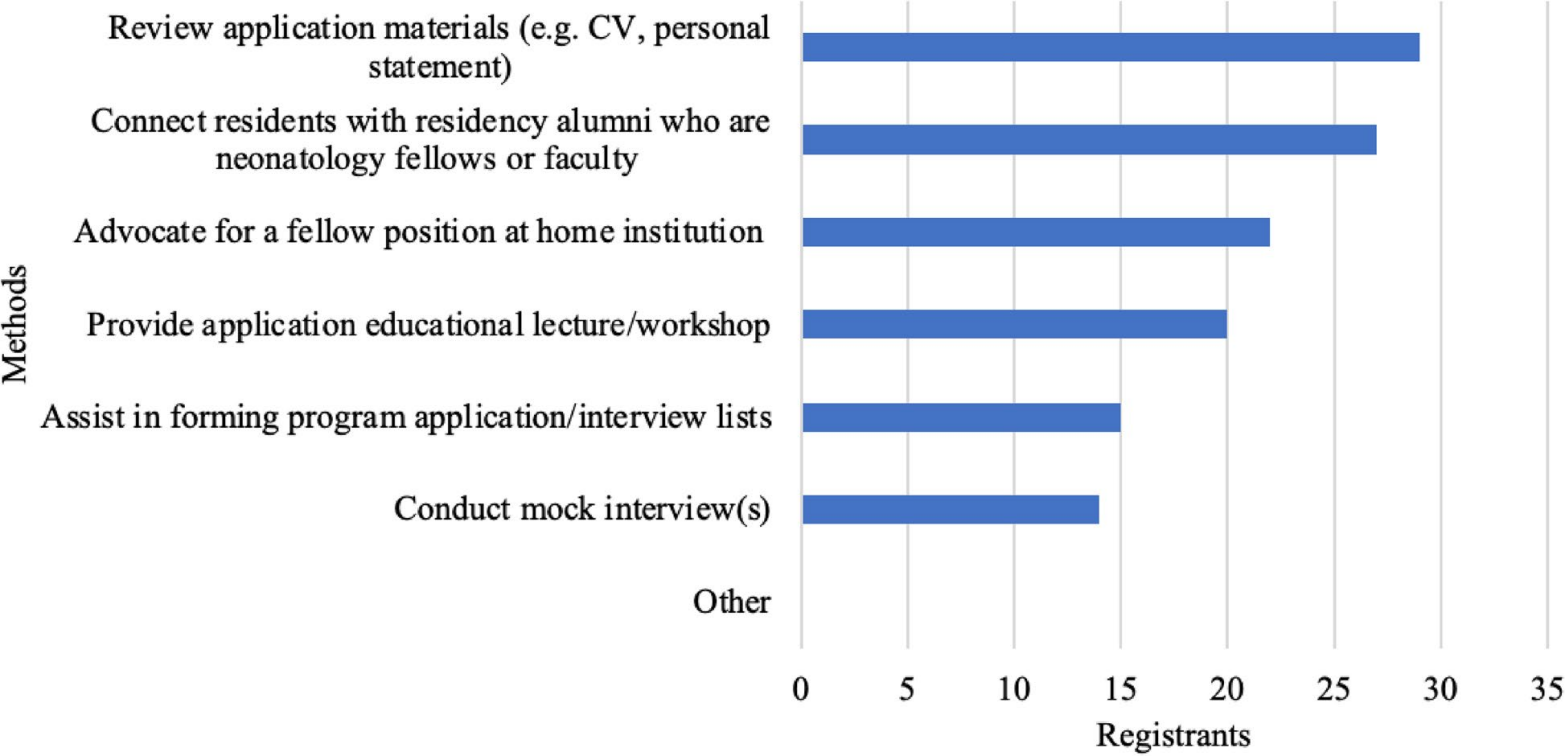
Specific methods of support that registrants were interested in having from their residency program

Our respondents applied to a median of 20 programs (IQR 14–35) for the 2022–2023 cycle resulting in a median of 13 interview invitations (IQR 9–16). Survey respondents reported they interviewed virtually at a median of 11 programs (IQR 8–15) and ranked a median of 11 programs (IQR 8–13). The most common factor influencing program rank list was geographic location (51%). Other important factors included interactions and impressions of program leadership (38%), program’s reputation (36%), and spouse/partner considerations (30%) (Fig. 2). Respondents were less interested in the opportunity to obtain an advanced degree (2%), size of the fellowship program (4%), and the fellowship call schedule (15%) (Fig. 2). Respondents indicated that they would desire support from their residency programs in the form of reviewing application materials (62%) and connecting with alumni in the field of neonatology (57%) (Fig. 3). The vast majority (40, 98%) of our respondents matched in the 2022–2023 cycle.

## Discussion

We describe a novel virtual curriculum to prepare trainees for applying to and interviewing for subspecialty fellowship, with the goal of increasing the transparency of the process surrounding this career step. We had 250 registrants in 2022 and there were 310 applicants for the NPM fellowship application cycle, suggesting these webinars likely captured a significant percentage of NPM fellowship applicants [1]. In conjunction with the majority of our respondents indicating they had little to no knowledge of the NPM application and interview process, this level of attendance highlights a currently unmet need for more specific and directive information for the applicant, a need that is likely not unique to NPM fellowship.

The virtual format allowed for flexibility in attendance, particularly with multiple options (live vs. video), allowing our information to reach residents across the country, regardless of resources, program, or location. Online views exceeded in-person attendance for every webinar, supporting that there is significant value to providing the curriculum asynchronously to increase the program’s reach.

Importantly, 26% of our registrants self-identified as URiM, compared to 14.2% of Neonatal-Perinatal Medicine fellows who identified as URiM in 2019, a decrease in fellow URiM representation since 2007 (Table 2) [9]. In 2022, approximately 1/3^rd ^of total applicants to NPM fellowship (total=333) identified as American Indian or Alaska Native (2), Asian (53), Black or African American (24), Hispanic, Latino, or of Spanish Origin (36), or Native Hawaiian or Other Pacific Islander (0) respectively [11]. This highlights the importance of accessible formal resources as a component of increasing diversity in the physician workforce particularly within the field of neonatology. In addition, we had over 30 panelists with varying gender, ethnicity, race, program size, and geographic location who provided broad viewpoints and multisource advice to applicants. Respondents also reported a large assortment of factors influencing their rank list likely indicating our webinars reached a diverse-set of applicants with distinctive career interests. While this webinar was not specifically designed for only URiM candidates, a future direction could include dedicated sessions for focused on recruitment of URiM fellows into neonatology.

Due to the limited availability of resources for specific subspecialty fellowship application processes, better understanding of trainee demographic interests and needs will help residency programs support their transitioning residents to fellowship. The majority of respondents indicated connecting residents with residency alumni in the field of neonatology and reviewal of application materials as desired support from their residency programs, illuminating possible future directions for residency programs to provide formalized fellowship application support to applicants (Fig. 3) [4].

Our webinar evaluations indicated these were high quality webinars and respondents were likely to recommend them to a peer. In addition, almost all of the respondents felt the webinars were helpful, demonstrating effectiveness in improving NPM fellowship applicants' comfort with the application and interview process (Table 3). The “Mastering the NPM Fellowship Application and Interview” webinar series is in its third year and requires minimal resources to produce, costing only administrative time and effort consisting of a few hours a month by the organizing committee (consisting of the moderators and faculty advisors) and the willing participation of our panelists. This would be very reproducible for other subspecialties across graduate medical education (GME) to create their own unique webinar series pertinent to their field’s fellowship application process.

Limitations to our study include low post-webinar survey response rates and subsequently respondent bias, which hinders the ability to generalize our results. In addition, our advertisement was through email, social media, and word of mouth, therefore we may have missed some of the potential applicant population who would be interested in registering for and attending our webinars.

Additional future directions of this webinar series will be based on participant feedback and program evaluation data. This webinar series will continue to be offered to NPM fellowship applicants on an annual basis for the foreseeable future. Future webinars will provide updated information as changes are made to the fellowship application by ERAS, such as geographic signaling, impact statements, and identification of significantly meaningful experiences.

## Conclusions

This webinar series serves to bridge the gap between fellowship applicants’ understanding of fellowship recruitment processes and fellowship program director expectations of applicants. The education provided was found to be helpful and of high quality. This webinar series is generalizable to other subspecialties across GME and can easily be replicated with minimal resources.

## Data Availability

The datasets used and analyzed during the current study are available from the corresponding author on reasonable request.

## Abbreviations

AAP: American Academy of Pediatrics
AΩA: Alpha Omega Alpha
ERAS: Electronic Residency Application Service
GME: Graduate Medical Education
NPM: Neonatal-Perinatal Medicine
NRMP: National Resident Matching Program
ONTPD: Organization of Neonatal-Perinatal Training Program Directors
PD: Program Director
REDCap: Research Electronic Data Capture (REDCap)
RPC: Residency Preparation Course
SMS: Specialties Matching Service
TECaN: Trainees and Early Career Neonatologists
URiM: Underrepresented in Medicine
USMLE: United States Medical Licensing Examination
